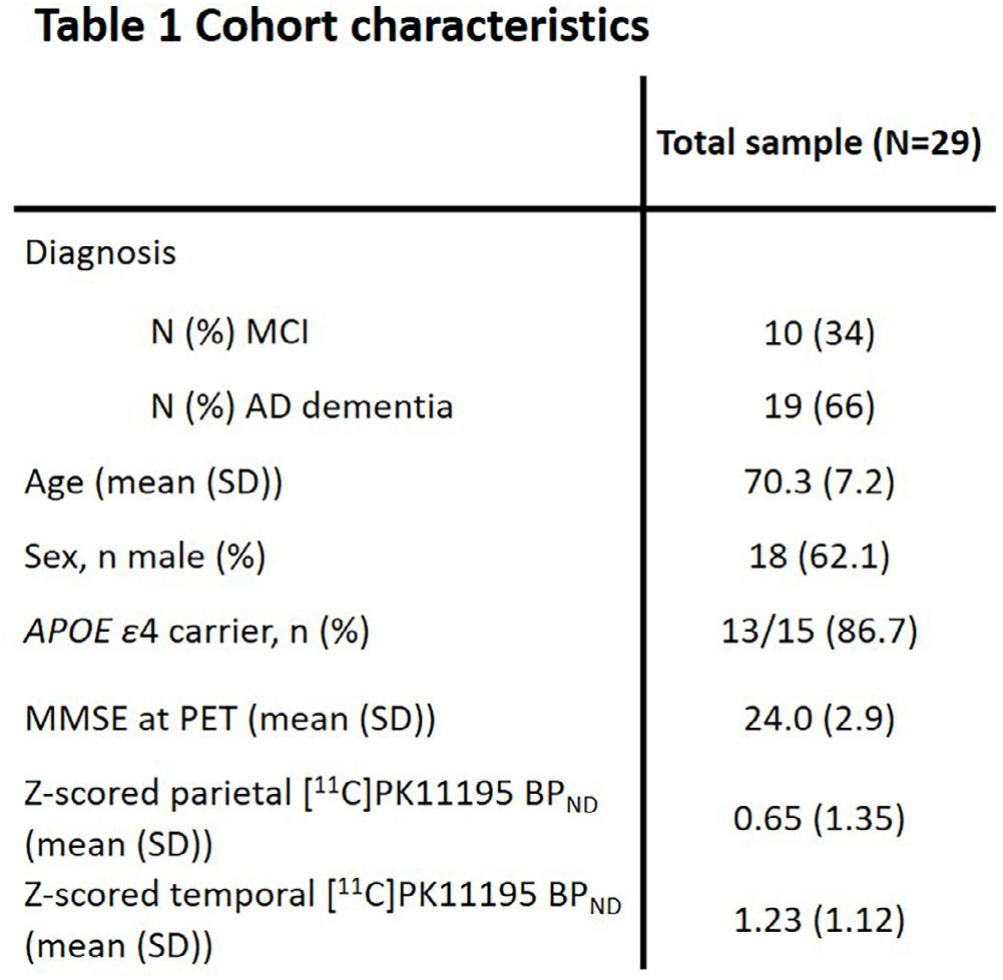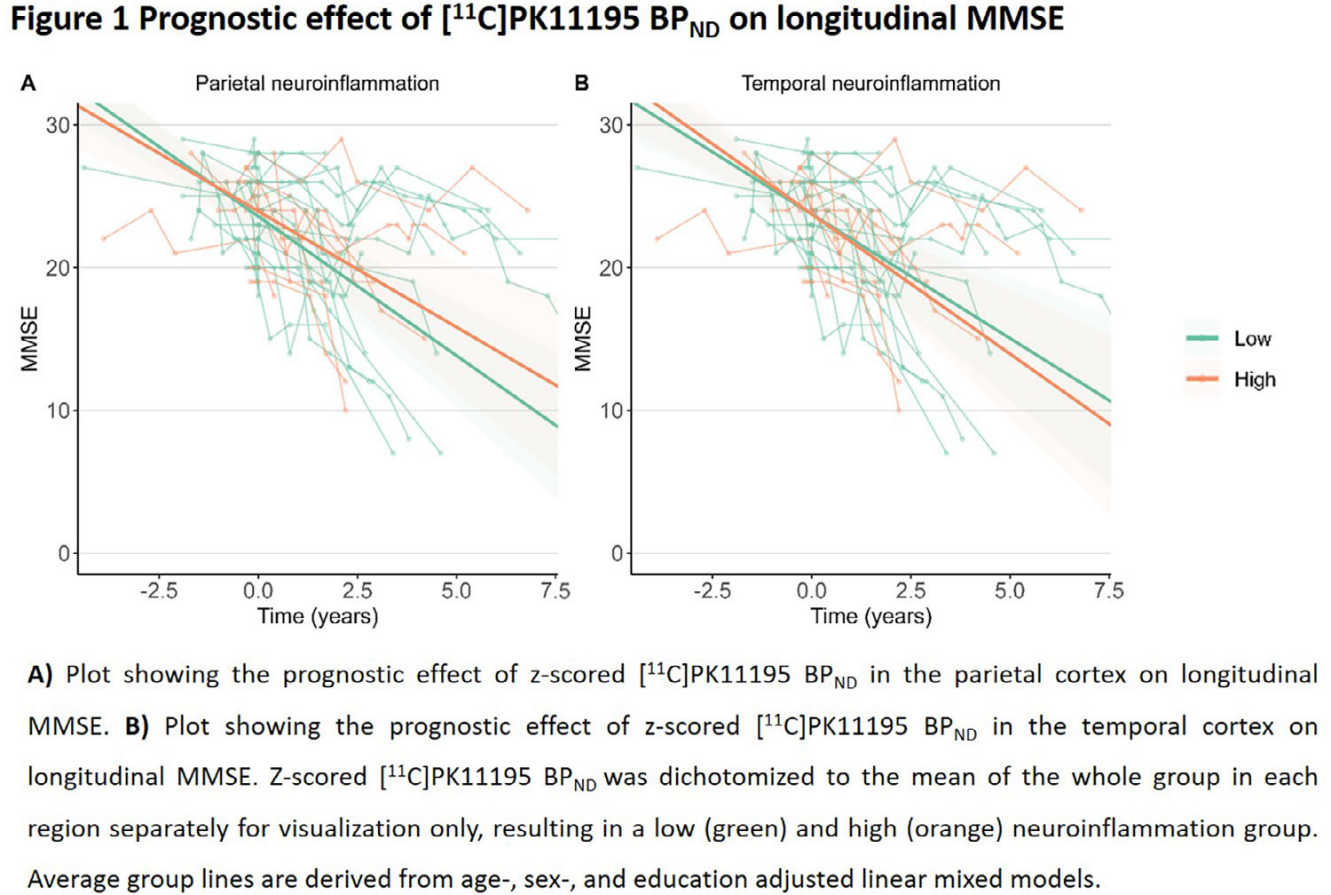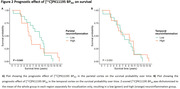# Prognostic value of neuroinflammation [^11^C]PK11195 PET on longitudinal cognitive decline and survival up to 15 years after PET in Alzheimer's disease

**DOI:** 10.1002/alz.087326

**Published:** 2025-01-09

**Authors:** Roos M. Rikken, Maqsood Yaqub, Emma M. Coomans, Anne E. van der Vlies, Frederik Barkhof, Albert D. Windhorst, Yolande A.L. Pijnenburg, Wiesje M. van der Flier, Ronald Boellaard, Everard G.B. Vijverberg, Elsmarieke van de Giessen

**Affiliations:** ^1^ Amsterdam Neuroscience, Brain Imaging, Amsterdam Netherlands; ^2^ Alzheimer Center Amsterdam, Neurology, Vrije Universiteit Amsterdam, Amsterdam UMC location VUmc, Amsterdam Netherlands; ^3^ Radiology & Nuclear Medicine, Vrije Universiteit Amsterdam, Amsterdam UMC location VUmc, Amsterdam Netherlands; ^4^ Amsterdam Neuroscience, Neurodegeneration, Amsterdam Netherlands; ^5^ Alzheimer Center Amsterdam, Amsterdam UMC, Amsterdam Netherlands; ^6^ Department of Radiology and Nuclear Medicine, Vrije Universiteit Amsterdam, Amsterdam University Medical Center, location VUmc, Amsterdam Netherlands; ^7^ University College London, London UK; ^8^ Alzheimer Center Amsterdam, Neurology, Vrije Universiteit Amsterdam, Amsterdam UMC, Amsterdam Netherlands; ^9^ Amsterdam Neuroscience, Neurodegeneration, Amsterdam, Noord‐Holland Netherlands; ^10^ Epidemiology and Biostatistics, Vrije Universiteit Amsterdam, Amsterdam UMC location VUmc, Amsterdam Netherlands

## Abstract

**Background:**

Neuroinflammation plays a key role in Alzheimer’s disease (AD) pathophysiology. However, whether neuroinflammation has a prognostic effect on disease progression is largely unknown. Therefore, we aim to investigate the role of neuroinflammation as measured using PET on longitudinal cognition and survival.

**Method:**

We included 29 amyloid‐positive participants (N=10 MCI, N=19 AD dementia) from a historical cohort who underwent dynamic [^11^C]PK11195 (TSPO) PET to quantify neuroinflammation (Table 1). [^11^C]PK11195 BP_ND_ was obtained using supervised cluster analysis and quantified in the parietal and temporal cortex. [^11^C]PK11195 BP_ND_ values were z‐scored to young healthy controls (N=12). Longitudinal MMSE covering a period up to 11 years was used to measure cognitive decline (median: 3.9, range: 0.3‐11.6 years). We used linear mixed models with random intercept and slope corrected for age, sex and education to investigate the effect of neuroinflammation on cognition. Survival data were available for all participants, up to 15.7 years after PET. To examine the influence of neuroinflammation on survival time, we used age, sex, and diagnosis adjusted cox proportional‐hazards models for both regions.

**Result:**

Demographic characteristics and mean [^11^C]PK11195 BP_ND_ can be found in table 1. Overall, the group showed a mean annualized decline of ‐1.86±1.42 points on MMSE and had a median survival time of 7.6 years (range: 0.4‐15.7) after PET. Temporal or parietal [^11^C]PK11195 BP_ND_ did not predict longitudinal MMSE (parietal: β=0.15, p=0.438; temporal: β=‐0.11, p=0.660) (Figure 1). Higher parietal [^11^C]PK11195 BP_ND_ was marginally associated with shorter survival (HR = ‐0.39, P=0.043) (Figure 2A). There was no significant difference in survival time with higher temporal [^11^C]PK11195 BP_ND_ (HR= ‐0.42, P=0.053) (Figure 2B).

**Conclusion:**

In this initial set of analyses covering up to 11 years of cognitive follow‐up and survival information of 15 years after PET, we found preliminary evidence of a shorter survival with increasing parietal neuroinflammation, but no associations between neuroinflammation PET and the rate of cognitive decline. Future research could explore the effect of neuroinflammation PET on more specific cognitive domains and biological measures such as cortical thickness.